# Impaired Tactile Temporal Discrimination in Patients With Hepatic Encephalopathy

**DOI:** 10.3389/fpsyg.2018.02059

**Published:** 2018-10-30

**Authors:** Moritz Lazar, Markus Butz, Thomas J. Baumgarten, Nur-Deniz Füllenbach, Markus S. Jördens, Dieter Häussinger, Alfons Schnitzler, Joachim Lange

**Affiliations:** ^1^Institute of Clinical Neuroscience and Medical Psychology, Medical Faculty, Heinrich Heine University Düsseldorf, Düsseldorf, Germany; ^2^Neuroscience Institute, Langone Medical Center, New York University, New York, NY, United States; ^3^Department of Gastroenterology, Hepatology and Infectious Diseases, Medical Faculty, Heinrich Heine University Düsseldorf, Düsseldorf, Germany

**Keywords:** behavioral, perception, somatosensory, liver cirrhosis, integration window

## Abstract

The sensory system constantly receives stimuli from the external world. To discriminate two stimuli correctly as two temporally distinct events, the temporal distance or stimulus onset asynchrony (SOA) between the two stimuli has to exceed a specific threshold. If the SOA between two stimuli is shorter than this specific threshold, the two stimuli will be perceptually fused and perceived as one single stimulus. Patients with hepatic encephalopathy (HE) are known to show manifold perceptual impairments, including slowed visual temporal discrimination abilities as measured by the critical flicker frequency (CFF). Here, we hypothesized that HE patients are also impaired in their tactile temporal discrimination abilities and, thus, require a longer SOA between two tactile stimuli to perceive the stimuli as two temporally distinct events. To test this hypothesis, patients with varying grades of HE and age-matched healthy individuals performed a tactile temporal discrimination task. All participants received two tactile stimuli with varying SOA applied to their left index finger and reported how many distinct stimuli they perceived (“1” vs. “2”). HE patients needed a significantly longer SOA (138.0 ± 11.3 ms) between two tactile stimuli to perceive the stimuli as two temporally distinct events than healthy controls (78.6 ± 13.1 ms; *p* < 0.01). In addition, we found that the temporal discrimination ability in the tactile modality correlated positively with the temporal discrimination ability in the visual domain across all participants (i.e., negative correlation between tactile SOA and visual CFF: *r* = −0.37, *p* = 0.033). Our findings provide evidence that temporal tactile perception is substantially impaired in HE patients. In addition, the results suggest that tactile and visual discrimination abilities are affected in HE in parallel. This finding might argue for a common underlying pathophysiological mechanism. We argue that the known global slowing of neuronal oscillations in HE might represent such a common mechanism.

## Introduction

The human brain constantly receives multiple signals from external sources through the senses. Precise neuronal processing of these signals and their temporal relationships is crucial for perception and behavior. If two signals arrive with sufficiently long temporal interval between both stimuli (stimulus onset asynchrony, SOA), they are readily perceived as two temporally separate events. However, the temporal resolution necessary to discriminate the two stimuli is limited and with decreasing SOA, subjects will perceive two stimuli only as one single stimulus with increasing probability. The threshold for which two stimuli can be successfully discriminated is altered in several diseases. For example, patients with motor impairments, such as Parkinson’s disease or dystonia, need longer time intervals to perceive two tactile stimuli as two separate events (Antelmi et al., 2017; Lee et al., 2018). This alteration has been assigned to impairments in the basal ganglia, which are believed to play a role in temporal perception (Lacruz et al., 1991; Pastor et al., 2004; Conte et al., 2016). Recent studies in healthy individuals additionally highlighted the role of primary somatosensory cortex (S1) for temporal perception of tactile stimuli (Hannula et al., 2008; Conte et al., 2012; Rocchi et al., 2016). In addition, Baumgarten et al. (2015, 2016) recently showed that neuronal oscillations in S1 correlate with temporal perception of tactile stimuli. Neuronal oscillations in the beta-band (∼15–20 Hz) predicted whether subjects perceived one or two stimuli. These studies suggested that neuronal oscillations in the beta-band of S1 form the basis of temporal perception in the tactile domain (Baumgarten et al., 2015, 2017a). In more detail, this model for temporal perception proposes that cycles of neuronal oscillations form temporal windows for neuronal integration of incoming information (see VanRullen, 2016 for a review). If these two stimuli fall into different cycles, they are processed separately and hence perceived as two separate stimuli. Previous studies suggest that in the somatosensory domain these integration windows are reflected in cycles of neuronal oscillations in the beta-band in S1 (Baumgarten et al., 2015, 2017a). Similarly, studies have proposed that such integration windows also exist in the visual modality and for audio-visual integration with cycles of the alpha rhythm (∼8–12 Hz) forming the temporal integration windows (e.g., VanRullen et al., 2006; Wutz et al., 2014; Cecere et al., 2015; VanRullen, 2016). These models of temporal perception state that temporal perception is mediated by the length of the cycles of neuronal oscillations. Consequently, if subjects show altered neuronal oscillations, these models would predict altered temporal perception.

In the present study, we studied tactile temporal perception in patients with hepatic encephalopathy (HE). HE patients are known to have slowed oscillatory activity (e.g., Butz et al., 2013) and thus are an ideal model to test the hypothesis that temporal tactile perception is mediated by discrete perceptual cycles in the beta-band. HE is a common complication in patients with liver cirrhosis and can serve as a model for slowed cortical oscillatory activity (Butz et al., 2013). In this patient population, the presence of liver cirrhosis restricts the detoxification function of the liver, which then leads to increased ammonia levels in the blood. The rise in ammonia levels are thought to lead to a low-grade cerebral edema, causing alterations in signal transduction, neurotransmission, and synaptic plasticity (Häussinger and Schliess, 2008; Prakash and Mullen, 2010; Felipo, 2013). Moreover, a slowing of oscillatory activity in visual and motor systems was observed (Timmermann et al., 2008; Kahlbrock et al., 2012; Butz et al., 2013; Götz et al., 2013). Likewise, slowed oscillatory activity was also reported for the somatosensory cortex of patients with HE (May et al., 2014). In the light of this works, it has been suggested that a global slowing of oscillatory activity spanning across the different cortical subsystems and across the different frequency bands forms a key mechanism underlying altered behavior and neuropsychiatric symptoms occurring in HE patients (Timmermann et al., 2008; Butz et al., 2013). Consequently, HE comprises a great variety of neuropsychiatric symptoms, including cognitive, vigilance, and motor impairments (Häussinger and Sies, 2013). Also the visual temporal perception is impaired in patients with HE, which is represented in a decreased critical flicker frequency (CFF; Kircheis et al., 2002). The CFF is defined as the specific frequency at which a flickering light that is presented with a decreasing frequency is first perceived as a discrete flicker. The CFF serves as an objective clinical parameter to detect and monitor HE. Moreover, decreases in CFF correlated with slowing of neuronal oscillations in the visual cortex (Götz et al., 2013; Baumgarten et al., 2018).

In summary, patients with HE show slowed oscillatory activity and impaired visual temporal perception. Based on the findings that demonstrated slowed oscillatory activity also in somatosensory cortex, we hypothesized in the present study that HE patients should also show impaired tactile temporal perception. We used an established paradigm to test temporal perception of tactile stimuli (Baumgarten et al., 2015, 2016, 2017a,b). Related to the slowed CFF in the visual system, we proposed that HE patients demonstrate slowed temporal perception in the tactile system and thus, need longer SOAs compared to healthy subjects to detect two separate stimuli.

## Materials and Methods

### Participants

Fifteen healthy controls (CON) and 16 patients (PAT) diagnosed with varying grades of HE due to liver cirrhosis participated in the experiment. Two PAT were excluded from analyses due to exclusively “1” reports regardless of SOA (see below for details). Three additional PAT were excluded from analyses due to unreliable fits of the behavioral data (see below for details). For details on the remaining 11 (14, respectively) PAT and 15 CON see Table 1.

**Table 1:** Characteristics of patient and control groups.

	Controls	mHE	HE
*N* (f/m)	15 (5/10)	5 (2/3)	6 (1/5) [9 (2/7)]
Age (y; median (first, third quartile))	65.0 (52.0, 69.8)	57.0 (46.0, 71.3)	64.5 (58.0, 75.0) [66.0 (60.8, 76)]]
CFF (Hz; median (first, third quartile))	42.6 (40.5, 43.3)	39.5 (37.6, 40.3)	36.7 (34.7, 37.9) [37.1 (34.7, 37.9)]
Stimulation amplitude (mA, median (first, third quartile))	3.2 (3.0, 4.2))	2.3 (2.0, 3.3)	3.4 (2.8, 3.8) [3.3 (2.7, 3.9)]
Etiology of cirrhosis	–	4 ALC, 1 overlap	4 ALC, 1NASH, 1 HCV, 1 CRYP, 1 NT, 1 AI

*Data are presented for those participants that entered the main analyses (see Figures 2, 3) because their behavioral data could be successfully fitted (see Supplementary Figure S2). The column HE shows additionally (in square brackets []) the data for all patients that successfully completed the task, e.g., including three patients that did not enter the main analyses due to non-successful fits (see Supplementary Figures S1, S2C). mHE, minimal hepatic encephalopathy; HE, manifest hepatic encephalopathy; CFF, critical flicker frequency; ALC, alcoholic; Overlap, overlap syndrome; NASH, non-alcoholic steatohepatitis; HCV, hepatitis C virus; CRYP, cryptogenic, NT, nutritive toxic; AI, autoimmune hepatitis.*

Patients were diagnosed with HE by means of clinical assessment in combination with the CFF (see below) and computer psychometry (Vienna test system, Dr. Schuhfried GmbH, Mödling, Austria). Computer psychometry tested for an age corrected skill set of cognitive, motoric, reaction time, and attention competencies.

Patients were categorized in two groups: (1) Minimal HE (labeled mHE), i.e., patients without overt clinical symptoms but lowered CFF and/or deficits in psychometric testing (Kircheis et al., 2002). (2) Manifest HE (labeled HE), i.e., patients with clinically visible symptoms of HE (e.g., tiredness, reduced attention, or flapping tremor), graded as HE1 (*n* = 7) or HE2 (*n* = 2) according to the *West-Haven-*Criteria, which are commonly used to classify patients with overt symptoms into four stages (Ferenci et al., 2002).

The CFF is typically used to detect patients with HE with a cutoff frequency of 39.0 Hz (Kircheis et al., 2002). In our study, three mHE patients showed a CFF > 39.0 Hz (39.5; 39.6; 42.2 Hz). Despite a CFF > 39.0 Hz, mHE was diagnosed in these patients by their deficits in the psychometric testing (Kircheis et al., 2002). Liver cirrhosis in all patients was confirmed by biopsy or Fibroscan/ARFI.

Exclusion criteria were psychiatric or neurological diseases apart from HE or abuse of alcohol or psychoactive drugs within the last 4 weeks. Also, patients with HE grade 3 or 4 were excluded from the study. All participants reported normal or corrected-to-normal vision and no tactile impairments. All patients were recruited from the Department of Gastroenterology, Hepatology and Infectious Diseases of the University Hospital Düsseldorf. All participants gave their written informed consent prior to the experiments. Healthy controls were financially reimbursed, patients received no financial reimbursement. The study was approved by the ethics committee of the University Hospital Düsseldorf (study no. 5779).

### Experimental Design and Paradigm

**FIGURE 1:**
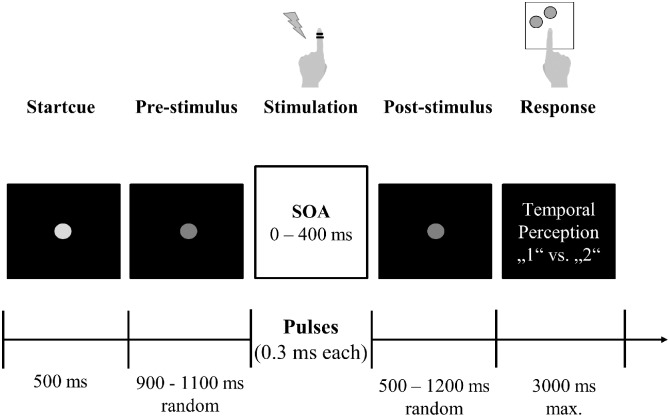
Experimental setup. Participants fixated a central gray dot. A decrease in luminance indicated the start of the stimulation period. After a jittered period of 900–1100 ms, participants received one or two electrical stimuli with varying SOA (0–400 ms) on their left index finger via ring electrodes. After another jittered period (500–1200 ms), visual response instructions were presented and participants reported their subjective perception (“1” vs. “2”) by button press with their right hand.

We adapted an established experimental task, which was designed to study tactile temporal perceptual discrimination in healthy humans (Baumgarten et al., 2015, 2016, 2017a,b). Participants were comfortably seated in a dimmed and sound-attenuated room. The start of every trial was signalized by a bright central fixation dot, serving as start cue (duration 500 ms; Figure 1). The following prestimulus period (duration randomized between 900 and 1100 ms) was indicated by a decreasing luminance of the cue. Next, the participants received either 1 or 2 short (0.3 ms) electrical pulses, applied by two ring electrodes placed at the distal phalanx of the left index finger. Electrical current was generated by a Stimulus Current Generator (DeMeTec GmbH, Langgöns, Germany). The amplitude of the pulses was adjusted individually to 150% of the subjective individual perception threshold. Subjective reports confirmed that stimulation at this level was clearly felt but below pain thresholds. The electrical pulses were applied with different SOAs ranging from 0 ms (i.e., only one stimulus was applied) to 400 ms with 12 steps in-between (15, 25, 35, 50, 100, 125, 150, 175, 200, 225, 250, and 300 ms). Next, the poststimulus period (duration randomized between 500 and 1200 ms) followed, during which only the fixation dot was visible. Durations of pre- and poststimulus epoch were randomized in every trial to reduce temporal expectation effects in the prestimulus period and motor preparation effects in the poststimulus period. The poststimulus period was followed by a written instruction, which marked the start of the response window (duration max. 3000 ms). Then, participants reported whether they perceived the stimulation either as 1 single or 2 temporally separated sensations, giving feedback by button-presses with their index or middle finger of the right hand. Button configurations were randomized between participants but kept constant within each individual. If no answer was given after 3000 ms or if participants responded before the instruction text was presented, a warning text appeared and the respective trial was discarded from analysis and repeated at the end of the block. After button press, the next trial started. The experiment was subdivided in blocks. Each block consisted of 50 trials. Between blocks participants had the chance to take a self-paced break of up to 2 min. All 14 SOAs were presented in a pseudo-randomized order. This pseudo-randomized order changed after each presentation of all 14 SOAs. Total duration of the experiment was limited to 30 min. Due to differences in reaction times and length of self-paced breaks this resulted in a varying total number of 350–450 trials (i.e., 7–9 blocks) per participant. Five patients ended the experiment earlier due to fatigue (duration of recorded data: ∼10–25 min, resulting in 100–300 trials). All controls finished the entire 30 min period.

Stimulus presentation was controlled using Presentation software (Neurobehavioral Systems, Albany, NY, United States). Each participant received instructions of the task but remained naïve to the purpose of the experiment and the different SOAs used. Standardized instructions on the task were given prior to the start of the experiment in form of an information sheet and verbal instructions, as well as in form of written instructions presented on screen. After instructions were given and before recording, every participant underwent a training phase of ∼5 min containing all possible SOAs to familiarize participants with the paradigm. Except the aforementioned warning text no further feedback was given during the actual test. Instructions and visual stimuli were presented via a projector on the backside of a translucent screen with a 60 Hz refresh rate positioned 60 cm in front of the participants.

Simultaneously to the behavioral study, we recorded neuronal activity with magnetoencephalography (MEG). The MEG data will be analyzed in future studies, in the present study we solely focus on the analysis of the behavioral data.

### Psychometric Fitting Function

As a measure for evaluating the individual tactile temporal discrimination abilities of each participant, we calculated the criticalSOA. The criticalSOA defines the specific SOA for which participants theoretically should exhibit a balanced response distribution (i.e., an equal amount of responses indicating a perception of one single stimulus and responses indicating two separate stimuli; Cecere et al., 2015; Baumgarten et al., 2017b). To account for potential lapse rates and response biases, we defined the criticalSOA as the SOA for which response rates reached the individual mean between the minimum and maximum mean response (Supplementary Figure S2).

To determine the criticalSOA of each participant, we fitted a sigmoid function to the individual raw behavioral data (Baumgarten et al., 2017b). Fitting procedure was conducted using the Palamedes toolbox for Matlab (Prins and Kingdom, 2009). As the independent variable we chose the SOAs (0, 15, 25, 35, 50, 100, 125, 150, 175, 200, 225, 250, 300, and 400 ms), whereas the average stimulus perception (averaged across trials, ranging from 1 to 2) at each SOA was chosen as the dependent variable. The fitting algorithm estimated four parameters of the logistic function: threshold, slope, guess rate, and lapse rate. We estimated the goodness of fit by computing the deviance and corresponding *p*-values. Only *p*-values >0.05 were estimated as a reliable fit of the experimental data and therefore included in further analysis (Supplementary Figure S2; Baumgarten et al., 2017b). For three PAT no reliable fit could be determined (Supplementary Figure S2C).

### Critical Flicker Frequency

The CFF is defined as the specific frequency at which a flickering light that is presented with a decreasing frequency is first perceived as a discrete flicker as opposed to a continuous light (Kircheis et al., 2002). The CFF was shown to be decreased in patients even with mild forms of HE, with a critical cut-off frequency of 39 Hz separating patients with HE from healthy controls (Kircheis et al., 2014; Barone et al., 2018).

Critical flicker frequency was assessed by an experienced psychologist (NDF) using the HEPAtonorm^TM^-Analyzer (NEVOlab, Maierhöfen, Germany) on the day of the tactile temporal perceptual discrimination task before experimental testing took place. The CFF was determined by presenting a flickering small red dot foveally with a starting frequency of 60 Hz. At this frequency, the flickering dot is always perceived as a constant light. Next, the frequency was decreased and subjects responded by button press as soon as they perceived the light as flickering. After standardized verbal instruction and a short training period, the CFF value was determined eight times per participant and the average value was taken as the individual CFF (see also Kircheis et al., 2002, 2014).

### Correlation Analysis, Effect Sizes, and Statistics

To test for significant differences in CFF, age, and electrical stimulation amplitudes between the three groups (controls, mHE, HE), we applied non-parametric Kruskal–Wallis tests. For *post hoc* pairwise comparisons and to test whether the criticalSOA differed across groups (CON and PAT; mHE, and HE), non-parametric Mann–Whitney *U*-tests were applied. From the resulting *z*-values effect sizes (*r*) were calculated:

$$r=\mathrm{abs}(\frac{z}{\sqrt{N}})$$

with N denoting the sample size (Fritz et al., 2012).

To analyze a potential correlation between the criticalSOA and the CFF, we computed the one-sided Pearson partial correlation coefficient between criticalSOA and CFF, controlling for age as a covariant, since the CFF is known to correlate with age (Kircheis et al., 2014). Additionally, we computed Pearson correlation coefficients within each group (controls and patients). 95% confidence intervals were estimated using bootstrapping approach (1000 repetitions). Correlation analysis was conducted in SPSS Statistics (IBM, Armonk, NY, United States).

All other statistical analyses were conducted in Matlab (Mathworks, Natick, MA, United States).

## Results

The following statistical tests are performed on only those 15 controls and 11 patients (5 mHE and 6 HE) that finally were included in the analyses (see section “Materials and Methods” and below for details on exclusion criteria).

A Kruskal–Wallis test revealed highly significant differences between groups (controls, mHE, HE, see Table 1) for the CFF [χ^2^(2) = 14.83, *p* = 0.0006]. *Post hoc* Mann–Whitney *U*-tests showed that the CFF significantly differed between controls and mHE (*z* = 2.36, *p* = 0.009; effect size *r* = 0.53), between controls and HE (*z* = 3.31, *p* = 0.0005; *r* = 0.51), and between mHE and HE (*z* = 1.83, *p* = 0.03; *r* = 0.55).

No significant differences between groups were found for age [χ^2^(2) = 1.14, *p* = 0.57; pairwise comparisons: all *p* > 0.37, *r* ≤ 0.25] and amplitude of the electrical stimulation [χ^2^(2) = 2.94, *p* = 0.23; pairwise comparisons: all *p* > 0.08, *r* ≤ 0.40].

### Behavioral Results and Fitting Procedure

Participants received one or two short electrical pulses with varying stimulus onset asynchronies (SOAs) to their left index finger (Figure 1). In a two-alternative forced choice tactile temporal discrimination task, they reported their subjective perception of the stimulation (“1” vs. “2” stimuli).

**FIGURE 2:**
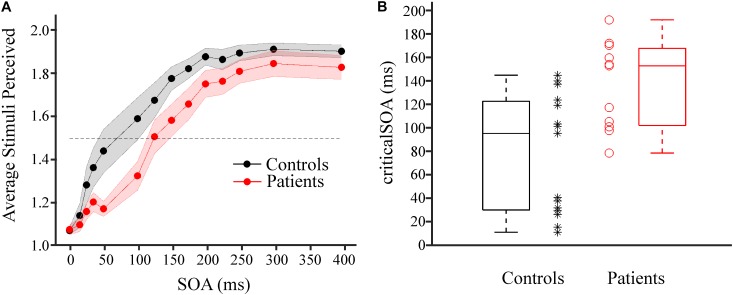
Results of tactile temporal discrimination task. **(A)** Average number of stimuli perceived as a function of the SOA between the two electrical stimuli for patient group (red, *n* = 11) and control group (black, *n* = 15). Shaded areas around dots indicate ±1 SEM. The dotted horizontal lines indicates mean perception rate of 1.5. **(B)** CriticalSOAs were determined individually (see Supplementary Figure S2). Box plots of the criticalSOAs and the individual criticalSOAs are presented for control participants (black box plot and black stars, *n* = 15) and for individual patients (red box plot and red circles, *n* = 11). Both groups differed significantly (*p* = 0.005, Mann–Whitney *U*-test).

On average, for both groups (PAT and CON) mean perception rates increased with increasing SOA (Figure 2A). To quantify the individual temporal discrimination abilities, we fitted a sigmoid function to the individual behavioral data and estimated from this curve the criticalSOA (see section “Materials and Methods” and Supplementary Figures S2A,B for details). Three patients (2 HE1 and 1 HE2) had to be excluded from further analysis due to unreliable fits (Supplementary Figure S2C). Supplementary Figure S1 illustrates the corresponding behavioral data with these three individuals excluded. Notably, these three patients exhibited low overall perception rates not reaching mean perception of 1.5 even for largest SOAs. In addition, two additional patients had been excluded from all analyses because they always responded “1,” regardless of SOA. Of these five patients, four belonged to the HE-group and only one belonged to the mHE-group.

Averaged across individuals, the median criticalSOA was 96.8 ms (first quartile; 31.4 ms, third quartile: 124.1 ms) for the CON group and 154.4 ms (first quartile; 103.5 ms, third quartile: 169.3 ms) for the PAT group (Figure 2B). Statistical analysis revealed a highly significant difference between both groups (*z* = 2.60, *p* = 0.005, *r* = 0.51). Additionally, we split the PAT group into mHE and HE patients and tested whether criticalSOAs differed between these groups. No significant difference was found between these groups (*z* = 0.46, *p* = 0.68, *r* = 0.14).

### Correlation of CriticalSOA and CFF

**FIGURE 3:**
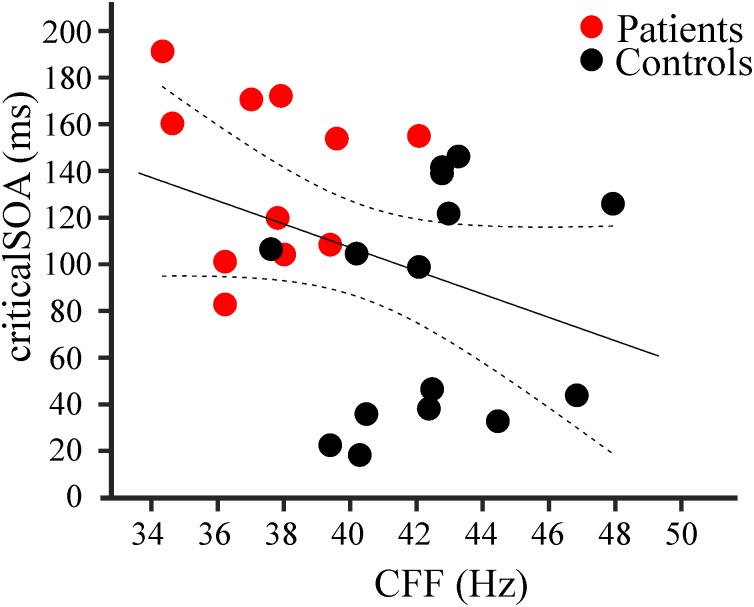
Results of correlation analysis. Negative correlation (*r* = –0.37; *n* = 26, *p* = 0.033) between individual criticalSOAs and individual CFF for patients (red dots, *n* = 11) and controls (black dots, *n* = 15). The black line represents the linear regression, the dotted lines the 95% confidence intervals for the mean.

Correlation analysis revealed a significant negative linear relationship between CFF and criticalSOA, corrected for age (*r* = −0.37, 95% confidence intervals: [−0.69, −0.05], *N* = 26, *p* = 0.033, Figure 3). That is, decreasing CFF is associated with increasing criticalSOA. This result indicates a positive correlation between visual and tactile temporal discrimination abilities.

Additionally, we computed correlation coefficients between CFF and criticalSOA, corrected for age, within each group (controls and patients). We did not find significant correlations for the group controls (*r* = 0.15, 95% CI: [−0.47, 0.64], *N* = 15, *p* = 0.62) nor for the group patients (*r* = −0.08, CI: [−0.75, 0.66], *N* = 11, *p* = 0.83].

## Discussion

In this study, we investigated the hypothesis that tactile temporal discrimination is impaired in patients with HE. To this end, HE patients and healthy controls received two subsequent electrical stimuli to their index finger with varying SOAs and had to report their subjective perception (“1” vs. “2” stimuli). We found that the SOA for which participants perceived the two stimuli as “2” in 50% of all trials and as “1” in the remaining 50% of all trials (denoted “criticalSOA”) was significantly prolonged in HE patients compared to healthy controls. The effect size of *r* = 0.51 indicates a strong effect (Fritz et al., 2012). In addition, we found that across all participants the criticalSOA correlated negatively with the CFF.

Patients with HE are known to reveal impairments in their visual temporal discrimination abilities. In particular, the CFF is slowed in HE patients compared to healthy participants and the CFF decreases with increasing severity of HE (Romero-Gómez et al., 2007; Torlot et al., 2013; Kircheis et al., 2014). Our results demonstrate that this disease-related impairment does also span the somatosensory modality, and particularly the temporal discrimination of tactile stimuli. This finding tallies with early work showing both sensory impairments on the behavioral level in HE (Brenner et al., 2015) and slowing of cortical oscillatory activity within the somatosensory system in this patient population (May et al., 2014). Moreover, the correlation of CFF and criticalSOA implies that the severity of the impairment of tactile temporal perception parallels the impairments of visual temporal perception. This implies a progression according to the clinical severity of HE.

We did not find a significant difference between the mHE group and the HE group. It should be noted, however, that five patients had to be excluded from analyses: either due to exclusive “1” reports or due to unreliable fits (with average perception <1.5 for the highest SOA, see Supplementary Figure S2C). Of these five patients, four belonged to the HE-group and only one belonged to the mHE-group. Thus, the non-significant result might partially be due to exclusion of the most severely impaired participants. In addition, both patients with the most severe HE (graded as HE2) were also strongly impaired in their tactile temporal perception so that they just reached an average perception of 1.0 (patient excluded from analysis) and 1.3 for the highest SOA. Despite the non-significant difference of the criticalSOA between mHE and HE groups, these results argue in favor of increased impairments in tactile temporal discrimination with increasing disease severity. Moreover, these results may reflect that the pathological mechanism underlying impaired tactile temporal perception already occurs in initial mild forms of HE. Other studies reported that mHE and HE groups significantly differ in terms of CFF (e.g., Kircheis et al., 2002; Oeltzschner et al., 2015). The most likely reason for the lack of difference might be the comparably small sample size especially in the HE2 group. For future studies in addition to increasing the number of severely impaired patients, we might also refine the parameters to differentiate between patient groups, e.g., by increasing duration of the SOAs further, so that also the most strongly impaired patients might be included.

One possible concern might be that our paradigm cannot differentiate whether the prolonged SOAs are caused by impairments on the sensory, decisional, or cognitive level. That is, patients’ prolonged SOAs might be due to impaired perceptual abilities, due to altered processes in the decision process (e.g., shifted decision criterions; see, e.g., Iemi et al., 2016; Limbach and Corballis, 2016) or cognitive impairments (patients simply did not understand the task). Notably, this concern would equally hold for the CFF. For example, the result that some patients predominantly reported “1” even for the largest SOA might be due to the fact that their criticalSOA was larger than 400 ms, or they had a strong bias toward reporting “1” or they did not understand the task and simply always pressed the “1” button. If patients did not understand the task, however, they might with equal probability have pressed always the “2” button, especially as the response buttons were counterbalanced across participants. A response pattern of always “2,” however, was never reported, speaking against impairment on a purely cognitive level. Also, some patients verbally reported after the experiment that they indeed simply always felt “1,” which might argue for a process on sensory rather than decisional level. Future studies are needed both in the visual and tactile modality to further elucidate the level of the impairments.

The correlation between impairments in visual (CFF) and tactile temporal discrimination (criticalSOA) suggests a common underlying mechanism across modalities. Recent studies proposed that temporal perception relies on discrete “perceptual cycles” mediated by cycles of neuronal oscillations (Baumgarten et al., 2015, 2017a; Cecere et al., 2015; VanRullen, 2016). These models postulate a cycle of a neuronal oscillation as the basic unit of temporal stimulus processing and perception. Two stimuli can only be perceptually distinguished if they fall into two separate cycles of a neuronal oscillation, while they will be perceptually fused to a single sensation if both stimuli fall within one cycle. Several studies have demonstrated that HE patients show slowed oscillatory activity in sensorimotor, visual, and somatosensory areas (Kullmann et al., 2001; Olesen et al., 2011; Butz et al., 2013; Götz et al., 2013; May et al., 2014; Baumgarten et al., 2018). According to the model of perceptual cycles, for slower oscillations, two stimuli are more likely to fall into one cycle. Thus, these patients should need longer SOAs to successfully discriminate two stimuli. Our results confirm this prediction on a behavioral level. In addition, studies found a correlation between parieto-occipital alpha oscillations and visual discrimination abilities (Götz et al., 2013; Baumgarten et al., 2018). To date, the direct mechanistic link between slowed somatosensory neuronal oscillations and impaired tactile temporal discrimination, however, is missing. Thus, it remains unclear whether similar pathophysiological processes underlie impaired visual and tactile discrimination. We did not find, however, significant correlations between CFF and criticalSOA within groups (patients and controls). This might be due to the low number of subjects entering the separate groups. On the other hand, for both groups, the correlation coefficient was close to zero, indicating that the correlation across all participants is mainly mediated by the groups. Similarly, Baumgarten et al. (2018) reported a significant correlation between CFF and alpha frequencies in visual cortex. This correlation was significant only across groups (HE patients and controls), but not within groups. These results indicate that correlations do not primarily rely on individual differences in CFF and criticalSOA. The individual measures might be too noisy or variable and reliable correlations can be detected only when taking larger intervals of the CFF and criticalSOA into account, i.e., by pooling controls and patients. Future analysis of the MEG data might provide further insights whether slowed neuronal oscillations represent the pathophysiological mechanisms underlying impaired tactile temporal discrimination in HE and linking it to visual impairments.

In addition to differences in prestimulus ongoing neuronal oscillations, also peri- or poststimulus effects might account for our results. For example, peri- or poststimulus phase resets might reset temporal integration windows (Wutz et al., 2014; Baumgarten et al., 2017a). In this view, stronger phase resets in controls compared to patients might lead to more consistent resets of integration windows and thus higher precision for temporal perception of subsequent stimuli. Again, future analysis of the MEG data might provide further insights in the neuronal mechanisms.

An alternative explanation for the impaired tactile temporal discrimination abilities might be found in the power of somatosensory alpha oscillations. Previous studies in healthy individuals reported that tactile temporal discrimination abilities correlate with prestimulus power of alpha oscillation (∼8–12 Hz) in somatosensory cortex, with higher alpha power leading to more “1” reports (Jones et al., 2010; Lange et al., 2012; Baumgarten et al., 2016; Craddock et al., 2017). Other studies suggested that alpha power modulates the decision criterion, with high alpha power biasing decisions to “missing” stimuli (Iemi et al., 2016; Limbach and Corballis, 2016). Increased power of alpha oscillations in HE patients might thus lead to more “1” reports. Indeed, some studies reported increased alpha power in HE patients, either in resting state activity in visual cortex (Götz et al., 2013) or in poststimulus activity in somatosensory cortex (May et al., 2014). However, none of the studies has linked somatosensory alpha power to tactile temporal perception in HE patients so far. Again, future analysis of the MEG data might help to disentangle the underlying pathophysiological mechanisms which might consist of one of the previous or a combination of both explanations.

Finally, it has been shown in numerous studies that attention influences perception. It seems therefore likely that attention also influences temporal perception. In fact, attention has been shown to rhythmically modulate perception and behavior (Landau and Fries, 2012; Song et al., 2014). In line with the abovementioned connection between temporal perception and oscillatory activity, several studies suggest that attention modulates neuronal oscillations (e.g., Calderone et al., 2014; Landau et al., 2015). However, in our present study, we did not explicitly modulate attention. In addition, HE patients seem to be specifically impaired in their visual and tactile temporal perception. Other perceptual abilities that are also affected by attention modulations seem less affected by HE. In sum, while we cannot exclude an influence of attention on our results, it seems unlikely to us that the impaired tactile temporal perception can be explained by attention alone.

In summary, we found that HE patients are significantly impaired in their tactile temporal discrimination abilities compared to a healthy control group. HE patients required a longer SOA between two tactile stimuli to veridically perceive them as two temporally separate events. To the best of our knowledge, this is the first study to extend findings of impairments of temporal perception in HE patients to the somatosensory domain. These behavioral results are in line with a model of discrete tactile temporal perception (Baumgarten et al., 2015, 2017a). Furthermore, we found that tactile temporal perception correlated with visual temporal perception, arguing for a global impairment in HE affecting the different sub-systems in parallel. While the behavioral results confirm predictions from previous models, further neuroscientific studies are needed to unravel the pathophysiological mechanisms underlying the impaired tactile temporal perception in patients with HE.

## Author Contributions

MB, TB, AS, and JL conceived the study. MB, TB, and JL designed the study. ML, TB, and JL collected the data. ML and JL analyzed the data and drafted the manuscript. N-DF and MJ recruited, tested, and categorized the patients. All authors critically revised the draft and approved the final version.

## Conflict of Interest Statement

DH belongs to a group of patent holders for the HEPAtonorm^TM^-Analyzer (device determining the critical flicker frequency).The remaining authors declare that the research was conducted in the absence of any commercial or financial relationships that could be construed as a potential conflict of interest.

## Supplementary Material

FIGURE S1 Same as Figure 2A, but including the patients for which the data could not be fitted (see Supplementary Figure S2C; controls: *n* = 15; patients: *n* = 14).

FIGURE S2 Results of the fitting procedure. **(A)** Psychometric functions were fitted to the individual mean responses as a function of SOA for the control group. Black horizontal lines indicate the criticalSOA, black vertical lines the corresponding SOA. **(B)** Same as panel **(A)**, but now for the patient group. **(C)** Individual mean responses for three individual patients for which the data could not be reliably fitted. These subjects were excluded from analyses and Figures 2, 3, but included in Supplementary Figure S1.
