# Supplementary figures and images for: Impaired Tactile Temporal Discrimination in Patients With Hepatic Encephalopathy
Source: Front Psychol. 2018 Oct 30;9:2059. doi: 10.3389/fpsyg.2018.02059 (PMC6218607; doi:10.3389/fpsyg.2018.02059)

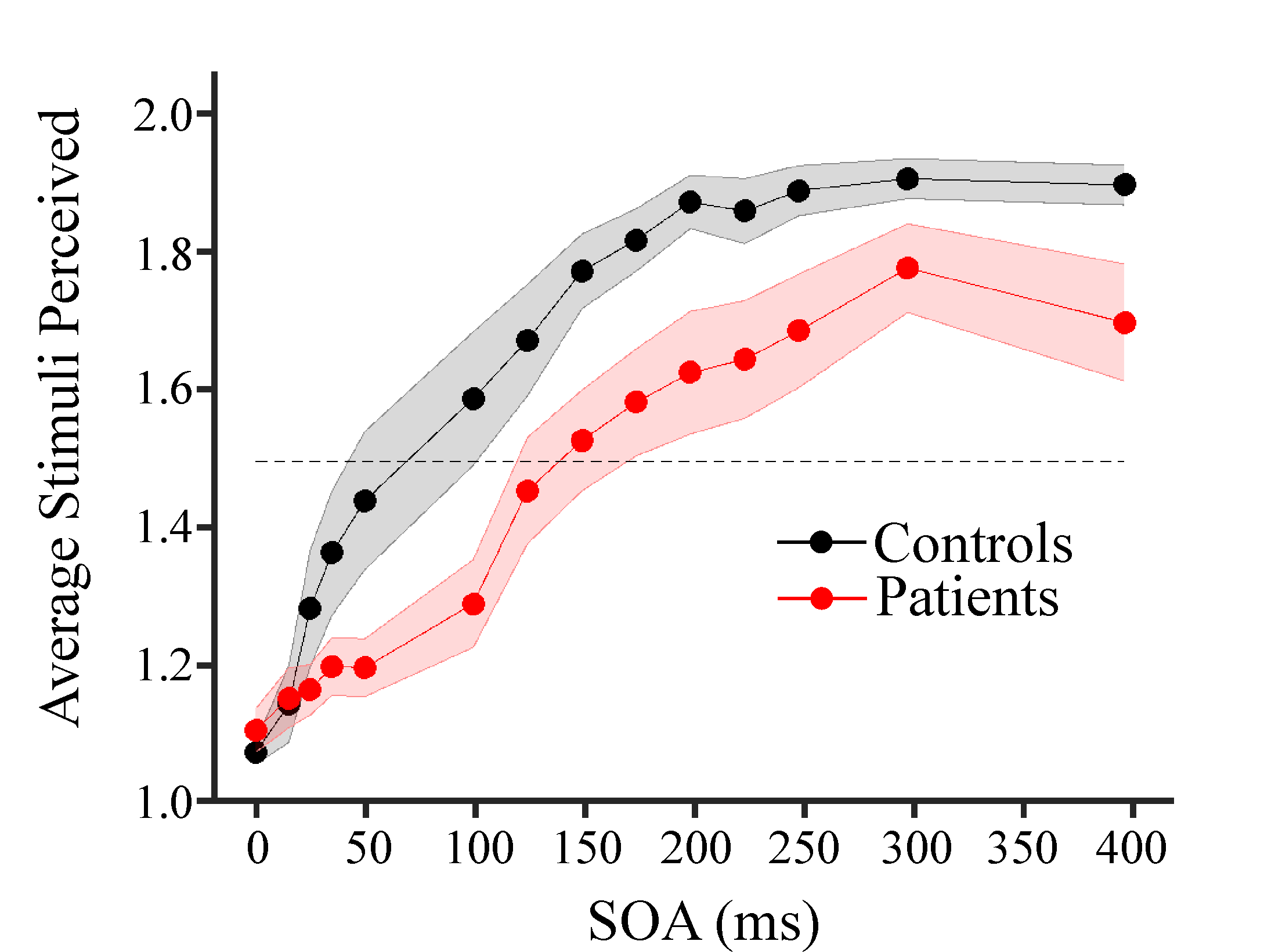

Supplement: FIGURE S1 — Same as Figure 2A, but including the patients for which the data could not be fitted (see Supplementary Figure S2C; controls: n = 15; patients: n = 14). [file Image_1.TIF]

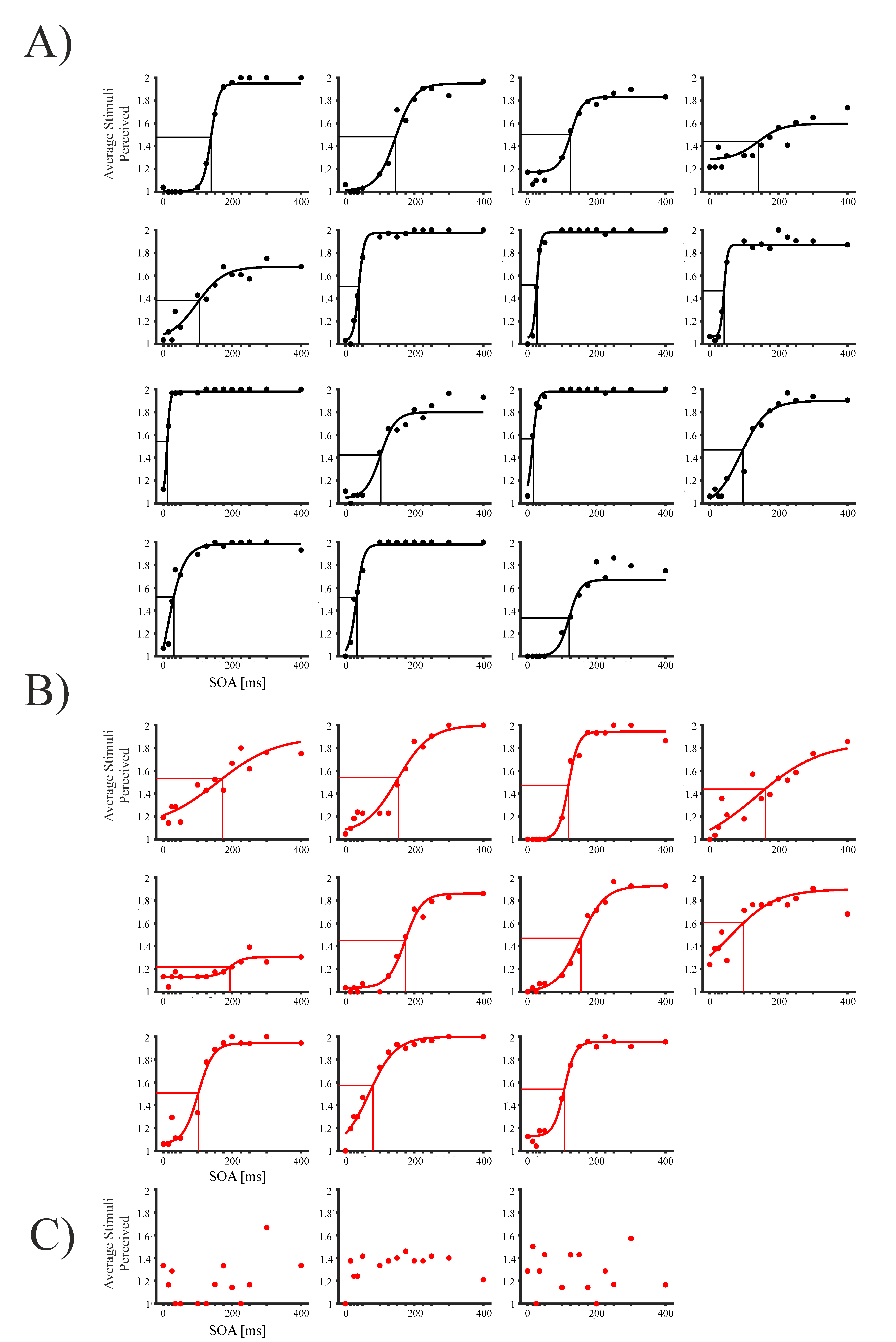

Supplement: FIGURE S2 — Results of the fitting procedure. (A) Psychometric functions were fitted to the individual mean responses as a function of SOA for the control group. Black horizontal lines indicate the criticalSOA, black vertical lines the corresponding SOA. (B) Same as panel (A), but now for the patient group. (C) Individual mean responses for three individual patients for which the data could not be reliably fitted. These subjects were excluded from analyses and Figures 2, 3, but included in Supplementary Figure S1. [file Image_2.TIF]
